# CT Angiography of the Head-and-Neck Vessels Acquired with Low Tube Voltage, Low Iodine, and Iterative Image Reconstruction: Clinical Evaluation of Radiation Dose and Image Quality

**DOI:** 10.1371/journal.pone.0081486

**Published:** 2013-12-05

**Authors:** Wei-lan Zhang, Min Li, Bo Zhang, Hai-yang Geng, Yin-qiang Liang, Ke Xu, Song-bai Li

**Affiliations:** 1 Department of Radiology, First Affiliated Hospital of China Medical University, Shenyang City, China; 2 Department of Clinical Epidemiology, First Affiliated Hospital of China Medical University, Shenyang City, China; 3 Institute of Radiology of China Medical University, Shenyang City, China; Villa Torri Hospital, Italy

## Abstract

**Objectives:**

We aimed to assess the effectiveness and feasibility of head-and-neck Computed Tomography Angiography (CTA) with low tube voltage and low concentration contrast media combined with iterative reconstruction algorithm.

**Methods:**

92 patients were randomly divided into group A and B: patients in group A received a conventional scan with 120 kVp and contrast media of 320 mgI/ml. Patients in group B, 80 kVp and contrast media of 270 mgI/ml were used along with iterative reconstruction algorithm techniques. Image quality, radiation dose and the effectively consumed iodine amount between two groups were analyzed and compared.

**Results:**

Image quality of CTA of head-and-neck vessels obtained from patients in group B was significantly improved quantitatively and qualitatively. In addition, CT attenuation values in group B were also significantly higher than that in group A (p<0.001). Furthermore, compared with the protocol whereby 120 kVp and 320 mgI/dl were administrated, the mean radiation dose and consumed iodine amount in protocol B were also reduced by 50% and 15.6%, respectively (p<0.001).

**Conclusions:**

With the help of iterative reconstruction algorithm techniques, the head-and-neck CTA with diagnostic quality can be adequately acquired with low tube voltage and low concentration contrast media. This method could be potentially extended to include any part of the body to reduce the risks related to ionizing radiation.

## Introduction

With increasing usage of computed tomography angiography (CTA) in recent years, more and more attentions have been paid to the potential risk of cancer related to radiation exposure during the procedures, especially to those patients who have chronic diseases and have to undergo examinations frequently for continuous treatment and monitoring [1]–[3]. In order to reduce this risk, the radiation dose should be kept as low as possible; meanwhile, an adequate image quality sufficient for making diagnosis should be maintained. Moreover, the adverse reactions caused by contrast material (CM) also possess some concerns to patients’ health [4], [5]. Among these concerns, acute renal failure is one of the notorious complications of procedures involving application of iodinated contrast media. For example, the contrast medium-induced nephropathy may account for about 12% of all cases of the nosocomial renal failure [6]–[8]. Therefore, prevention of such a nephropathy is crucial as it is associated with an increased mortality rate [9], [10]. Since the adverse reactions are associated with the effectively consumed iodine amount, a small amount of CM should be used in scanning protocol to minimize the impairment of patients, when applicable [11].

The method whereby low tube voltage with reduced radiation dose and effective iodine amount are employed has been tested in several studies [12]–[14]. Since the mean photon energy of 80 kVp in the x-ray beam moves closer to the k-absorption edge of iodine (33.2 keV) [15]–[19] when compared with 120 kVp, a higher vascular enhancement can be satisfactorily acquired with the increased iodine absorption at a reduced radiation dose level. However, the clinical implementation of such a method was limited due to increased noise and artifacts, which can potentially affect the accuracy of diagnosis [13], [20].

Previously, iterative reconstruction algorithm was introduced and proved to be useful for reducing the quantum noise compared with the filtered back projection (FBP) reconstruction algorithm [21], [22]. Many studies have demonstrated that iterative reconstruction algorithm has the potential to preserve the diagnostic acceptability in low-dose CT examinations [22], [23], however, a disadvantage of artificial oversmoothing image appearance with a waxy texture appears to be inevitable [21], [24]. Recently, adaptive iterative dose reduction 3D (AIDR 3D) algorithm, which is a newer-generation iterative reconstruction algorithm, becomes available for clinical use. AIDR3D processes image data with various models and utilizes FBP images as baseline images in every iteration. Several reports suggest that this new iterative reconstruction algorithm greatly improves the spatial resolution and produces images more naturally with reduced noise [24], [25].

In the present study, we tested the hypothesis that AIDR 3D could yield images with diagnostic quality for the head-and-neck vessels when low tube voltage and low concentration contrast media are used.

## Materials and Methods

### Ethics Statement

The present study was approved by the institutional review board of China Medical University. The written informed consents were obtained from all the participants prior to CTA procedures.

### Patient Population

A pilot test was performed on 4 healthy volunteers (3 males and 1 female, age was 27, 48, 51 and 68, respectively and BMI was 27.4, 26.5, 26.3 and 24.6, respectively) to validate our protocols prior to commencing the studies on the patients enrolled in this study. Two radiologists with more than 10 years of experiences were involved and concurred that the method did provide the quality of image sufficient for making clinical diagnosis.

From November 2012 to January 2013, 107 consecutively referred patients for the head and neck CTA were selected for this study. Patients with severe renal impairment (serum creatinine>2 mg/dl) or having an allergic reaction to CM, pregnant and lactating women were excluded from examination. Among 107 patients, twelve patients with hemorrhage and infarction that destroyed the intracranial vessels following surgical procedures, and additional three patients with movements during scanning were also excluded. The remaining 92 patients were randomly divided into two group: group A and group B: 46 patients in group A (25 males and 21 females, age 55.3±8.6, BMI 24.3±3) had their head-and-neck CTA scanned with 120 kVp. 46 patients in group B (24 males and 22 females, age 56.9±10, BMI 23.9±3) were scanned with 80 kVp. The demographic information of patients in the present study was summarized in Table 1.

**Table 1:** Patient Demographics and Scanning Parameters.

	Protocol A	Protocol B	P
Number of patients	46	46	
Male/Female	25/21	24/22	0.83
Age(y)	55.3±8.6	56.9±10	0.42
Body Weight(kg)	67±8.7	67.8±12.4	0.72
BMI(kg/m^2^)	24.3±3	23.9±3	0.5
Scan length(cm)	36.2±2.1	35.8±2.3	0.43
Concentration of contrast media (mgI/ml)	320	270	

BMI: Body mass index, calculated as weight (kg)/(height in m)^2^.

### CT Scanning and Contrast Medium Infusion Protocols

All examinations were performed with a 320-row CT scanner (Aquilion ONE Dynamic Volume CT; Toshiba, Tokyo, Japan) with the following settings: beam collimation, 160*0.5 mm; rotation time, 0.5 s; pitch, 0.869; section thickness and intervals, 0.5 mm and 0.4 mm; image matrix, 512*512; and field of view, 240 mm. An automatic tube current modulation program (Auto mA, Toshiba, Tokyo, Japan) was used in both groups with a noise index of 10 HU recommended by the manufacturer for the head-and-neck CTA. The tube current at 120 kVp was in the range of 100–500 mA, while it was from 178 to 500 mA at 80 kVp. The helical scan started from the carina of trachea to the top of the head; the scan length depended on the height of patients. Automatic bolus-tracking program (Toshiba, Tokyo, Japan) was used to monitor changes in region of interest (ROI) intensity and trigger the scanning when a threshold was reached. In 120 kVp protocol, we began the real-time monitoring of ROI at the same time when contrast material was injected. Since the time to peak of CT value arrives slightly faster in 80 kVp than that in 120 kVp, a 3 second delay after contrast material injection was added to synchronize the time to reach the peak value of CT scan. The scanning commenced when the triggering threshold of 150UH was reached in the descending aorta. The whole scanning was completed in 3 seconds.

The CM was injected with an 18-gauge intravenous catheter through the right elbow vein by using a high pressure syringe (Ulrich Missouvi XD, Ulm, Germany). The amount of contrast was determined according to the equation: amount = (10+scan time)*flow rate. The flow rate was determined as follows: 5 ml/s for BW<80 kg, 5.5 ml/s for BW between 80 kg and 100 kg, 6 ml/s for BW>100 kg. This injection method was verified and recommended by the technologists and had been employed in our department as a standard procedure. The following two contrast media were used in the present study: Ioversol 320 (Optiray 320 mgI/ml, Tyco, Quebec, Canada) was selected for its high concentration in conventional protocol with 120 kVp (protocol A), while Iodixanol 270 (Visipaque 270 mgI/ml, GE Healthcare, Cork, Ireland)was chosen for its low concentration in the protocol with 80 kVp (protocol B). The delivery of CM was immediately followed by an injection of 50 ml chasing saline with the same injection rate. The images in protocol A were reconstructed with FBP while those in protocol B were reconstructed with AIDR 3D. We chose the standard level of ADIR 3D, which allows a maximum of 75% noise reduction based on the CT manufacturer’s recommendation and our own experience. All images were transformed to a workstation (Vitrevitral Station System; Ziosoft, Tokyo, Japan) for further analysis.

### Radiation Dose Assessment

The dose-length product (DLP) and computed tomography dose index (CTDI) displayed on the CT system were used to calculate the radiation dose.

### Quantitative Images Assessment

**Figure 1:**
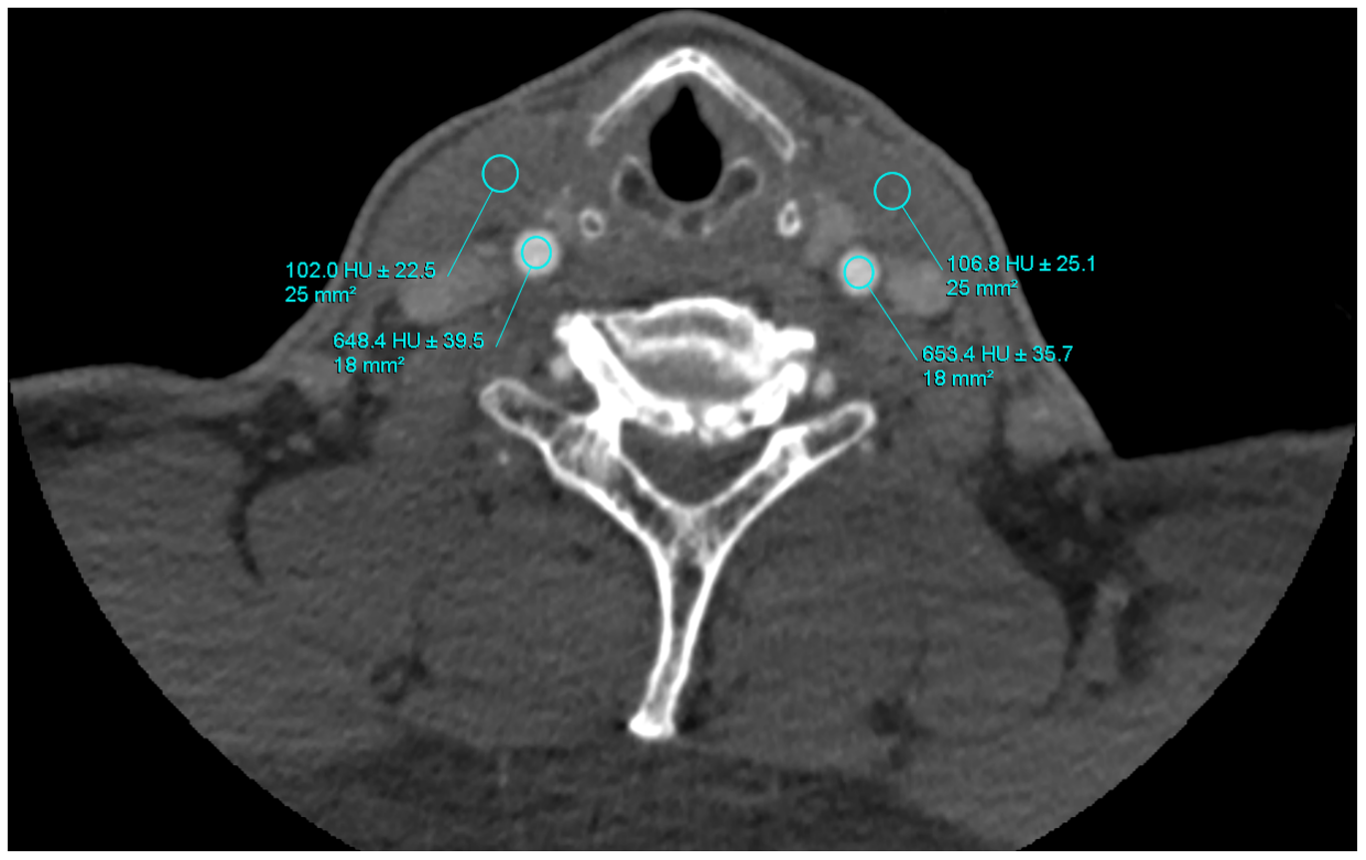
Determination of image noise, vascular attenuation. The CT attenuation value was measured at circular regions of interest (ROI) placed in the center of common carotid arteries. Data were shown as mean ± standard deviation. Additional ROIs were placed at the sternocleidomastoid muscle adjacent to the vessel contour.

The CT attenuation value (C_V_) was measured at circular ROI, placed in the center of the vessels at the following sites: ascending aorta (around the carina of trachea), bilateral common carotid arteries (around the ventricular bands), bilateral internal carotid arteries (around the glottis), and bilateral middle cerebral arteries. On the four levels above, additional ROI (25 cm^2^) was placed at the pectoralis major muscle, sternocleidomastoid muscle, longus scapitis and cerebral tissue, respectively. The CT attenuation value of the additional ROIs (C_A_) was used to calculate the contrast-to-noise ratio (CNR). All vessel ROIs were made as large as possible according to vessel sizes but should avoid vessel wall, calcifications or metallic artifacts to prevent partial volume effects. Image noise (N) was defined as the mean standard deviation of the attenuations of the vessels computed from aforementioned four positions. CNR was determined by the equation: CNR = (C_V_−C_A_)/N (Fig. 1).

### Qualitative Images Assessment

Image quality was rated on axial, curved planar reconstructions and volume renderings by two radiologists who have with more than 10 years of experience in assessing the head and neck imaging and were blinded to all scanning and processing conditions. We evaluated vessel images of significant segments (D>1.5 mm) for graininess, vessel sharpness, streak artifact and the overall image quality with a 4-point scale. Image graininess was graded as following: 4, excellent with small and homogeneous graininess; 3, good; 2, acceptable; 1, unacceptable with excess grain. Vessel sharpness was graded as following: 4, sharpest; 3, good; 2, suboptimal; 1, blurry. Streak artifact was graded as following: 4, none or minimal artifact; 3, artifacts occupying parts of the image, but not interfering with diagnostic decision making; 2, artifacts occupying the entire image, but diagnosis still possible; 1, unable to evaluate, severe artifact makes diagnosis impossible. The overall image quality was graded as following: 4, excellent; 3, good; 2, acceptable; 1, undiagnosable. The image with 1 point was considered as unacceptable. In case of inter-observer disagreement, the final decisions were reached by consensus.

### Statistical Analysis

All statistical analyses were performed with SPSS version 17.0 software (SPSS, Chicago, IL, USA). Data were shown as mean ± standard deviation unless otherwise noted. We looked for statistically significant differences in the radiation dose and image quality measurements between protocol A and protocols B. As we found in experiments, quantitative image quality measurement and radiation dose conformed normal distribution; therefore, they were subjective to 2-tailed independent sample t test. Qualitative image quality measurement conformed skew distribution and it was subjective to Mann-Whitney U test. The enumeration data was calculated with Chi-square test to study the differences. For all statistical analyses, p<0.05 was considered significant. Inter-observer agreement for image quality was performed with Kappa test, which was interpreted as poor (k<0.40), moderate (0.4≤k<0.75) and good (k≥0.75).

## Results

### Patient Demographics

There was no significant difference between protocol A and protocol B with respect to age, sex, body weight, BMI or scan length (p>0.05, Table 1).

### Radiation Dose

As presented in Table 2, mean overall CTDI was 50% lower for protocol with 80 kVp (3.9 mGy±0.5 vs 7.8 mGy±0.9, p<0.001). The DLP was significantly lower in the 80-kVp protocol (p<0.001), leading to an effective dose reduction of 50% (189.5 mGycm±26.4 vs 379 mGycm±48.1, p<0.001).

**Table 2:** Radiation Dose.

	Protocol A	Protocol B	P
CTDI (mGy)	7.8±0.9	3.9±0.5	<0.001
DLP(mGy*cm)	379±48.1	189.5±26.4	<0.001

CTDI: computed tomography dose index, DLP: dose length product, ED: effective dose.

### Image Quality

For quantitative images assessment, the mean CT attenuation values for the ascending aorta, bilateral common carotid arteries, bilateral internal carotid arteries and bilateral middle cerebral arteries were significantly higher for protocol B than those for protocol A (p<0.001 for each layer, Table 3). Mean image noise was lower in vessels from protocol B than that in protocol A (37.5 HU±5.2 vs 40.4 HU±5.3, p = 0.011). The CNRs for the four positions were significantly higher in protocol B than those in protocol A (p<0.001).

**Table 3:** Quantitative image quality.

	Protocol A	Protocol B	P
CT number(HU)			
Ascending aorta	421.8±61.1	598.1±85.8	<0.001
Common carotid arteries	553.3±91.5	735.7±124.6	<0.001
Internal carotid arteries	531.5±87.2	681.6±99.9	<0.001
Middle cerebral arteries	485.1±69.1	611.5±99.6	<0.001
Image noise(HU)	40.4±5.3	37.5±5.2	0.11
CNR			
Ascending aorta	8.6±1.9	13.7±1.7	<0.001
Common carotid arteries	11.2±2.9	16.6±2.8	<0.001
Internal carotid arteries	11±2.5	15.6±2.5	<0.001
Middle cerebral arteries	11±2.2	15.1±2.6	<0.001

CNR: Contrast-to-noise ratio.

Four patients were excluded from measurements of internal carotid arteries and middle cerebral arteries due to the occlusion of lumens in protocol B.

**Figure 2:**
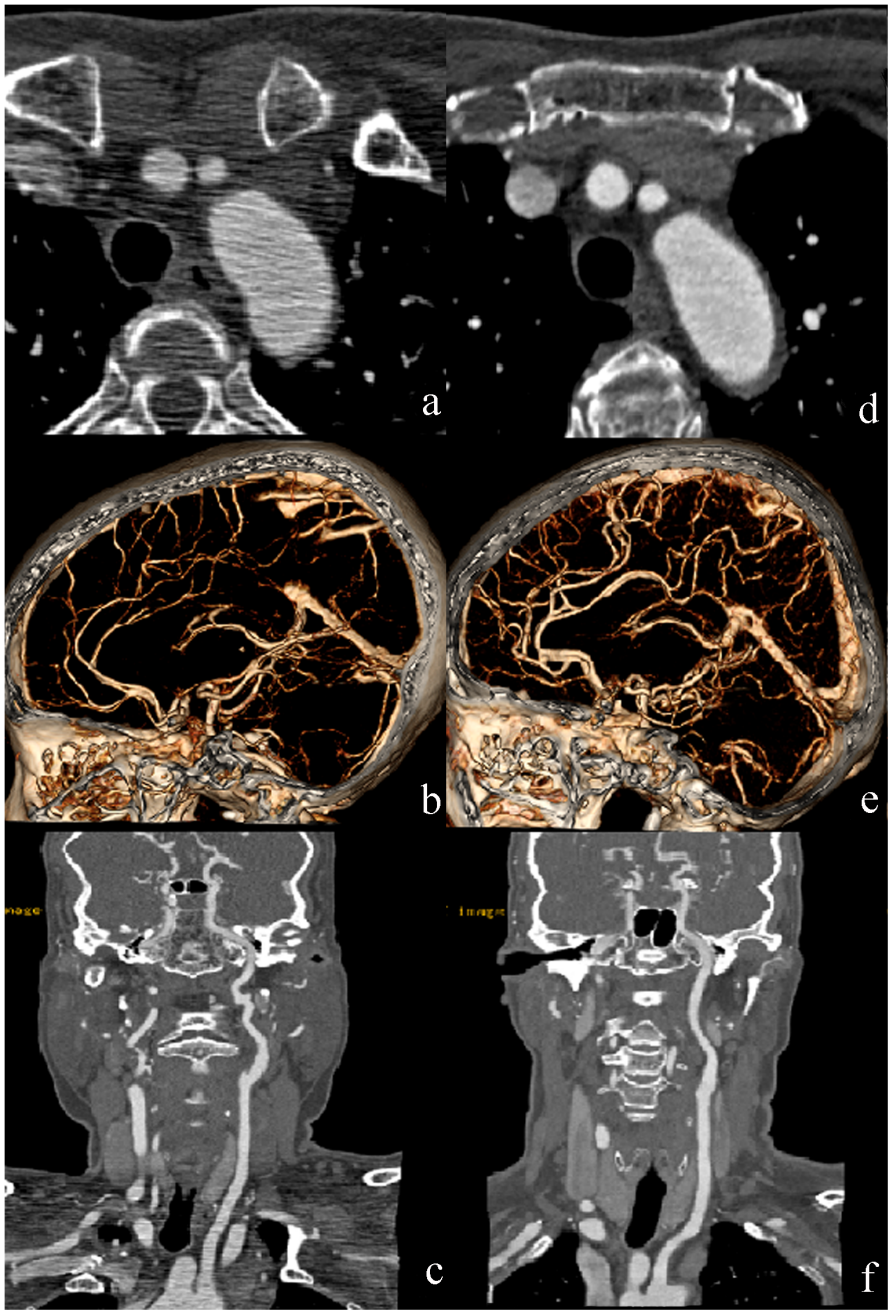
The images compared between two protocols. (a–c) CT images in a 64-year-old woman (BM, 62 kg; BMI, 26.84) in protocol A. (d–f) CT images in another 64-year-old woman (BM, 68 kg; BMI, 26.56) in protocol B. (a) and (d) axial images; (b) and (e) volume rendered images; (c) and (f) curved planar reconstructed images. The images in protocol B with iterative reconstruction decreased the streak artifacts and noises without losing the vessel sharpness and image quality.

In a qualitative evaluation, all the images were sufficient for rendering clinical diagnosis possible. There was no statistically significant difference in vessel sharpness (p = 0.59) and overall image quality (p = 0.74) between two protocols. However, there were statistically differences with respect to image graininess (p<0.001) and streak artifact (p<0.001) in favor of protocol B (Table 4, Fig. 2). The inter-observer agreement was good with respect to graininess (k = 0.82) but moderate to vessel sharpness, streak artifact, and overall image quality (k = 0.61, 0.61 and 0.73, respectively).

**Table 4:** Qualitative image quality.

	Protocol A	Protocol B	P
	(Percentiles, P_25_, P_75_)	(Percentiles, P_25_, P_75_)	
Grassiness	(2,2)	(3,3)	<0.001
Vessel sharpness	(3,4)	(3,4)	0.59
Streak artifact	(2,3)	(3,3)	<0.001
Overall image quality	(3,3)	(3,3)	0.74

## Discussion

In the present study, we found that a 50% reduction in the radiation dose and higher CT attenuation of vessels can be achieved by using 80 kVp and low concentration CM, which are consistent with those reported by Faggioni et al [26]. When combined with AIDR 3D technique, the improved imaging of head and neck arteries were sufficient for clinical diagnosis. At a level of 75%, iterative reconstruction algorithm could offer significantly lower noise and higher CNR without decreasing the sharpness. Furthermore, such a combination of low radiation dose, low CM and iterative reconstruction algorithm significantly improve the image quality on 80 kVp protocol with respect to graininess and streak artifacts. The results are in accordance with those reported by Yamada et al [24] and Ohno et al [25]. To our knowledge, this study represents the first one of its kind about CTA of the head-and-neck vessels with low tube voltage and low iodine; it thus could be of an important significance for clinical use, especially for the patients with thyroid disorders in which radiation damage possesses severe consequences of thyroid function.

With the development of techniques, numerous dose reduction strategies have been implemented, including tube voltage reduction, fixed tube current reduction, automatic exposure control, high helical pitch and scan length optimization [27]. One reason why we chose the low-tube-voltage technique is that it drastically reduces the radiation dose, which is proportional to the square of the tube voltage [28], while the tube current correlates to dose in a linear fashion [29]. Our study shows that the required dose is 50% lower in 80 kVp-protocol than that in 120 kVp protocol and these results are similar to those reported by Nakaura et al [30] and Oda et al [13], in which the tube voltages were also reduced from 120 kVp to 80 kVp. However, a different result was also reported by Namimoto et al [31] regarding to dose reduction, in which a 28.8% dose reduction was achieved when switching from 120 kVp to 80 kVp. The reason for the discrepancy between our findings and Namamoto’s was probably due to the different methods employed: In his study, an automatic exposure control in combination with decreasing tube voltage was used. In our study, we combined the automatic tube current modulation program with low tube voltage to further minimize radiation dose exposure. This technique adjusts the tube current by monitoring of tissue attenuation and real-time adjustment (z-modulation) of the base tube current as a function of the projection angle, with a delay of 360° [32], [33]. Compared with fixed tube current, the automatic tube current modulation not only reduces the radiation dose but also provides the similar level of image noise, which potentially improves diagnostic acceptability [32]. Although the tube current is slightly higher when the voltage decreases from 120 kVp to 80 kVp at the same noise index (in our study, it changes from 100–500 mA to 178–500 mA), it has little impact on the reduction of radiation dose [34]. Furthermore, the combination of low tube voltage and automatic tube current modulation was proved to be very efficient to reduce the dose as demonstrated and discussed in several similar studies [33], [35].

It is well known that there is a dose-dependent association between the iodine CM and contrast-induced nephropathy, which in turn is associated with increased morbidity and mortality [10]. Although many studies have proposed the prevention of contrast-induced nephropathy, the practical clinical settings for minimization of iodine amount have remained unchanged [8], [9], which are mainly due to the fact that a reduction in the effective iodine amount may attenuate the enhancement of vessels and consequently adversely affect diagnostic accuracy [36], [37]. By using low tube voltage technique, a better vascular enhancement can be achieved with less iodine amount. We prefer to decrease the iodine amount by using a low concentration CM, the osmolarity of which is also lower than that of CM used in protocol A. Lower osmolarity causes lesser injury to patients without affecting the image quality [4], [5]. In our study, despite a 15.6% reduction of mean effective iodine amount was achieved in protocol B(270 mgI/ml), the degree of contrast enhancement was significantly greater than that in protocol A(320 mgI/ml) in all anatomic structures examined (p<0.001).

Our study also possesses several limitations. Firstly, all the patients enrolled in the present study have a body weight between 49–95 kg. Because of the combined effect of photon deficiency and beam hardening [38], low-tube voltage techniques tend to decrease the image quality of the heavier patients. It is thus necessary to perform more studies on those people with a relative larger size. Secondly, we did not test the diagnostic accuracy; only the quantitative and qualitative image quality of the protocol with low concentration CM and low tube voltage were assessed. In the absence of DSA of most patients, the evaluation of disease diagnosis such as arteriostenosis and aneurysm needs further study. Thirdly, the reduction of CM is limited as we prefer to lower the iodine volume by decreasing the concentration rather than the total amount of CM [39]. In our study, the CT attenuation values of protocol B are significantly higher than those in protocol A, indicating that a concentration lower than 270 mgI/ml can be used in the 80-kVp protocol [36]. Lastly, the reduction of radiation is limited as the results of our study show attenuated noise and CNR when 80 kVp was used. Further study is warranted to explore this matter.

## Conclusion

In conclusion, the 80 kVp protocol with low concentration contrast media can dramatically decrease the radiation of the head-and-neck CTA with improved image quality by using AIDR 3D.
